# Supplementary material for: Human Alpha 1 Antitrypsin Suppresses NF-κB Activity and Extends Lifespan in Adult Drosophila
Source: Biomolecules. 2022 Sep 22;12(10):1347. doi: 10.3390/biom12101347 (PMC9599126; doi:10.3390/biom12101347)
Supplement: Supplementary file 1 [file biomolecules-12-01347-s001.zip › biomolecules-1896648-supplementary.pptx]

## Slide 1
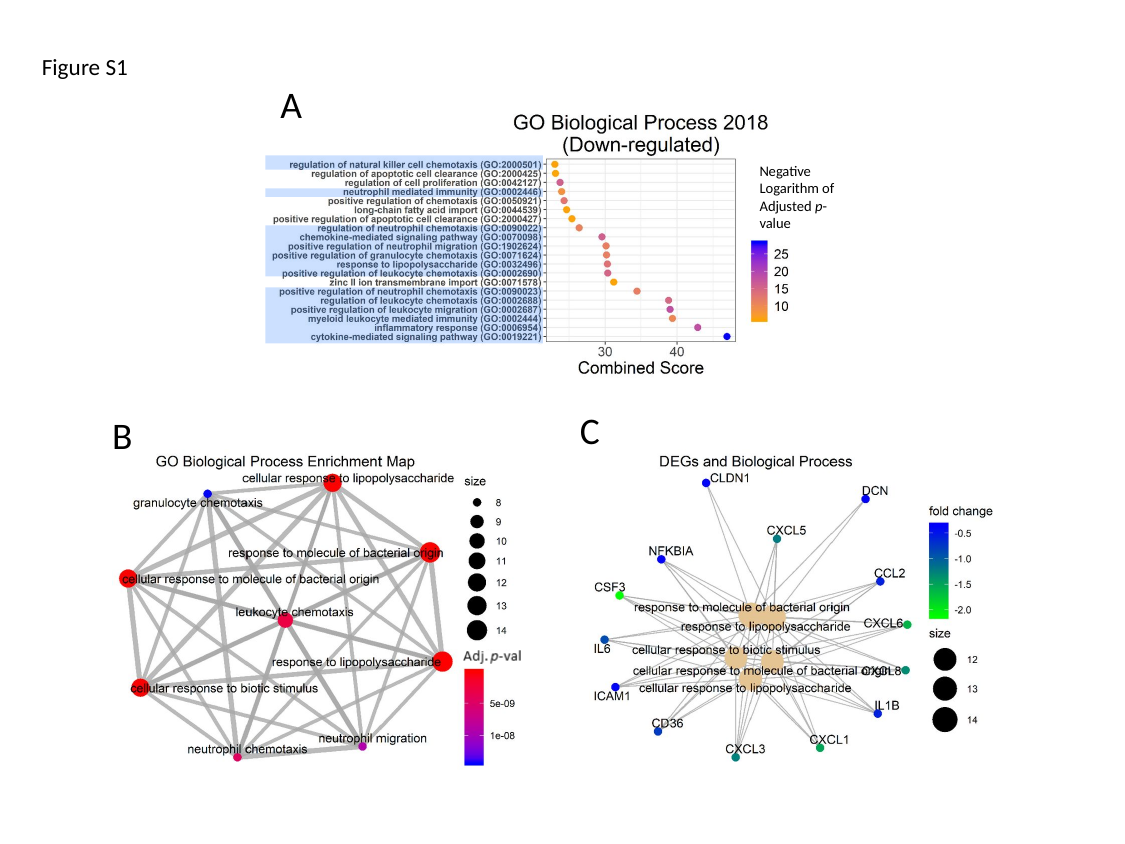

Figure S1
A
Negative Logarithm of Adjusted p-value
C
B

## Slide 2
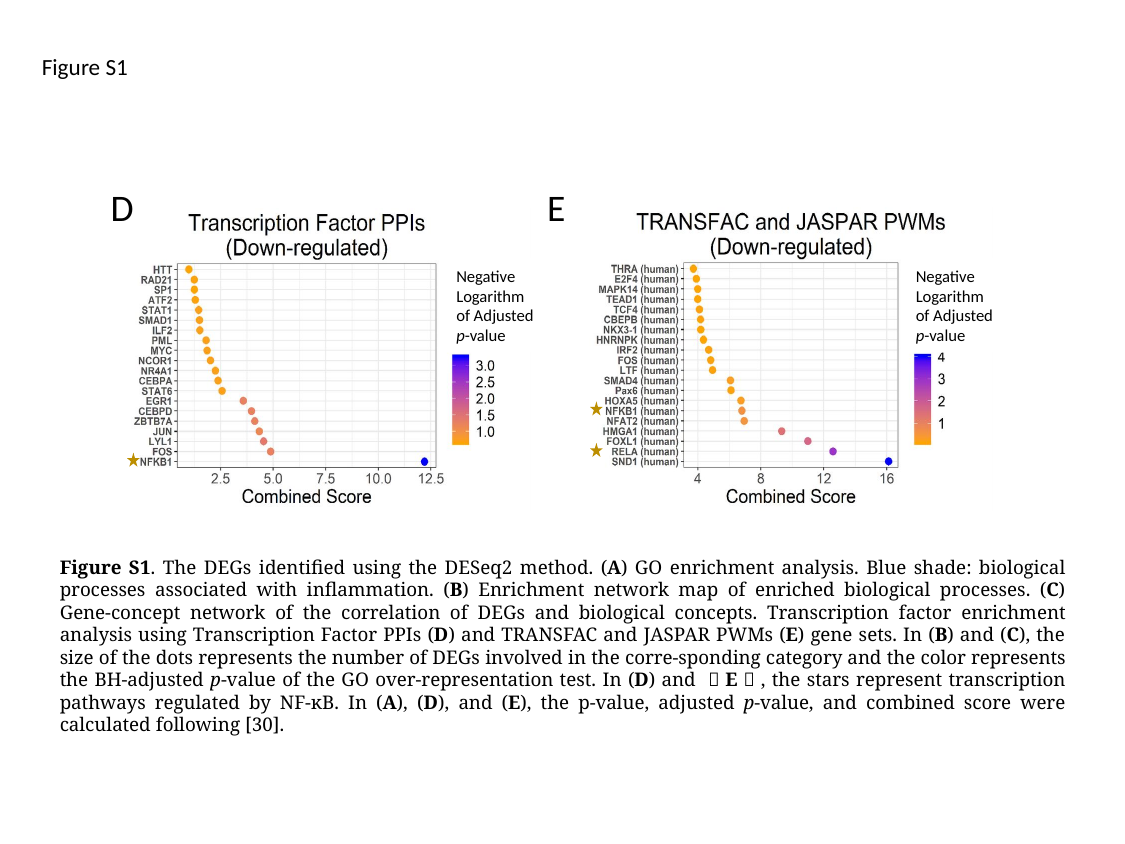

Figure S1
D
Negative Logarithm of Adjusted p-value
E
Negative Logarithm of Adjusted p-value
Figure S1. The DEGs identified using the DESeq2 method. (A) GO enrichment analysis. Blue shade: biological processes associated with inflammation. (B) Enrichment network map of enriched biological processes. (C) Gene-concept network of the correlation of DEGs and biological concepts. Transcription factor enrichment analysis using Transcription Factor PPIs (D) and TRANSFAC and JASPAR PWMs (E) gene sets. In (B) and (C), the size of the dots represents the number of DEGs involved in the corre-sponding category and the color represents the BH-adjusted p-value of the GO over-representation test. In (D) and （E）, the stars represent transcription pathways regulated by NF-ĸB. In (A), (D), and (E), the p-value, adjusted p-value, and combined score were calculated following [30].

## Slide 3
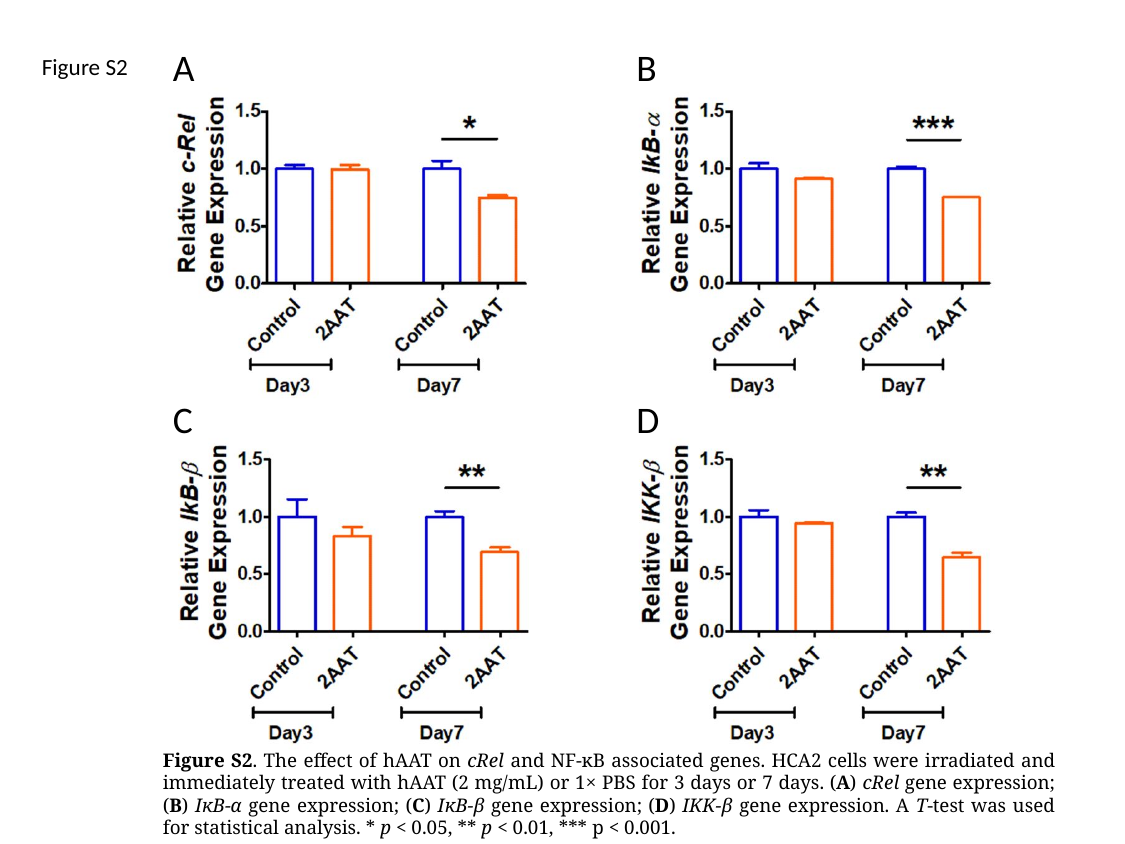

A
B
C
D
Figure S2
Figure S2. The effect of hAAT on cRel and NF-ĸB associated genes. HCA2 cells were irradiated and immediately treated with hAAT (2 mg/mL) or 1× PBS for 3 days or 7 days. (A) cRel gene expression; (B) IĸB-α gene expression; (C) IĸB-β gene expression; (D) IKK-β gene expression. A T-test was used for statistical analysis. * p < 0.05, ** p < 0.01, *** p < 0.001.

## Slide 4
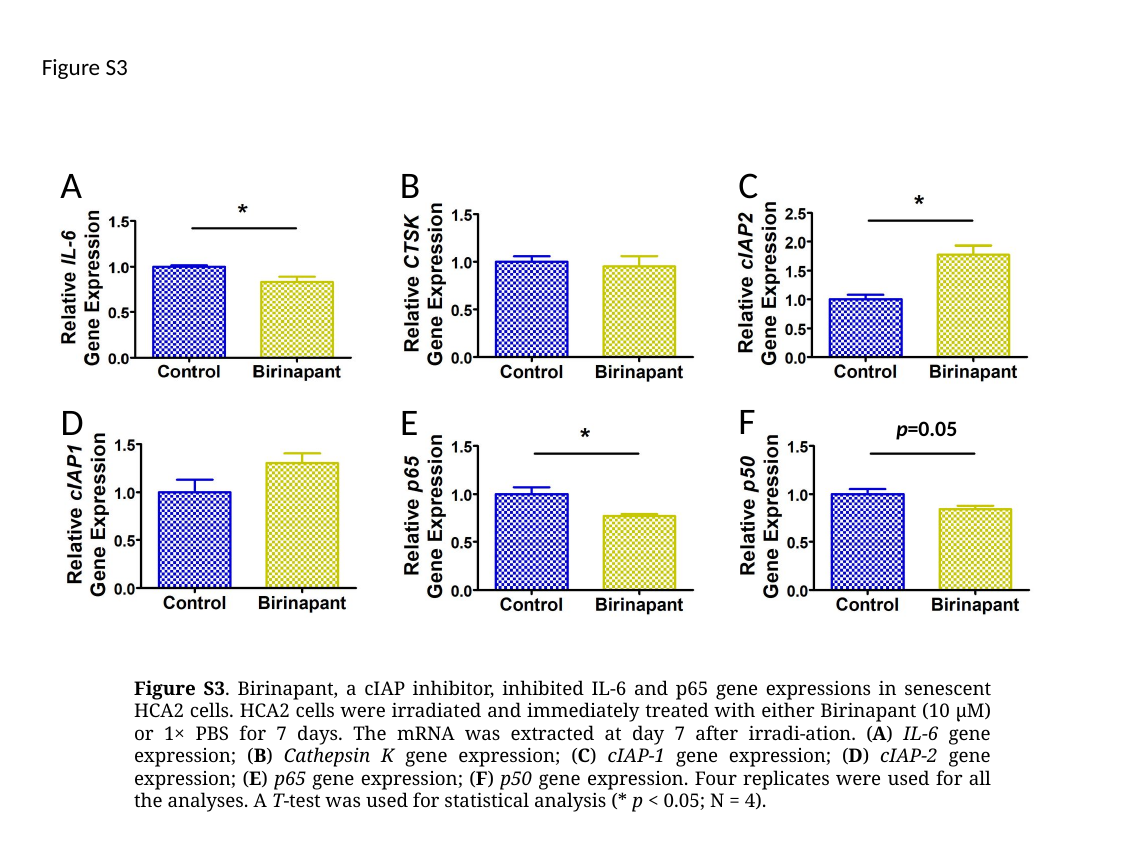

Figure S3
A
C
B
F
D
E
p=0.05
Figure S3. Birinapant, a cIAP inhibitor, inhibited IL-6 and p65 gene expressions in senescent HCA2 cells. HCA2 cells were irradiated and immediately treated with either Birinapant (10 µM) or 1× PBS for 7 days. The mRNA was extracted at day 7 after irradi-ation. (A) IL-6 gene expression; (B) Cathepsin K gene expression; (C) cIAP-1 gene expression; (D) cIAP-2 gene expression; (E) p65 gene expression; (F) p50 gene expression. Four replicates were used for all the analyses. A T-test was used for statistical analysis (* p < 0.05; N = 4).

## Slide 5
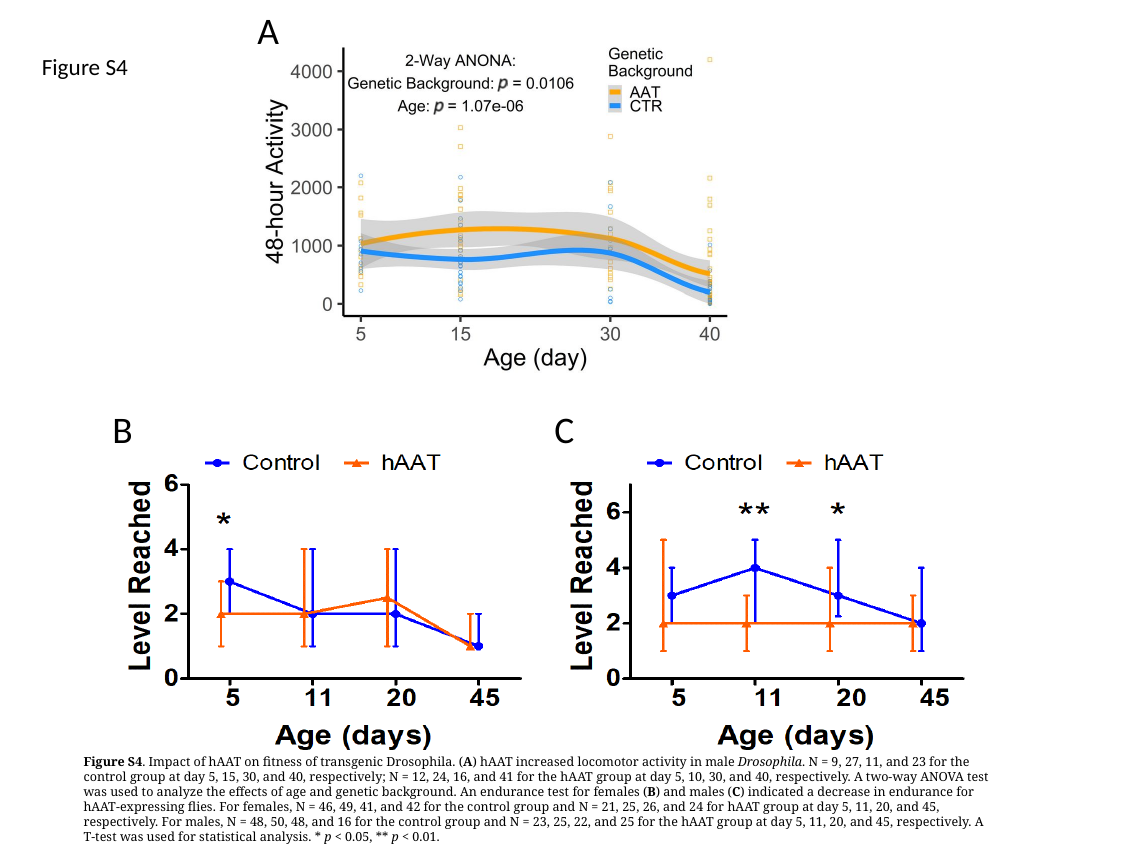

A
C
B
Figure S4
Figure S4. Impact of hAAT on fitness of transgenic Drosophila. (A) hAAT increased locomotor activity in male Drosophila. N = 9, 27, 11, and 23 for the control group at day 5, 15, 30, and 40, respectively; N = 12, 24, 16, and 41 for the hAAT group at day 5, 10, 30, and 40, respectively. A two-way ANOVA test was used to analyze the effects of age and genetic background. An endurance test for females (B) and males (C) indicated a decrease in endurance for hAAT-expressing flies. For females, N = 46, 49, 41, and 42 for the control group and N = 21, 25, 26, and 24 for hAAT group at day 5, 11, 20, and 45, respectively. For males, N = 48, 50, 48, and 16 for the control group and N = 23, 25, 22, and 25 for the hAAT group at day 5, 11, 20, and 45, respectively. A T-test was used for statistical analysis. * p < 0.05, ** p < 0.01.

## Slide 6
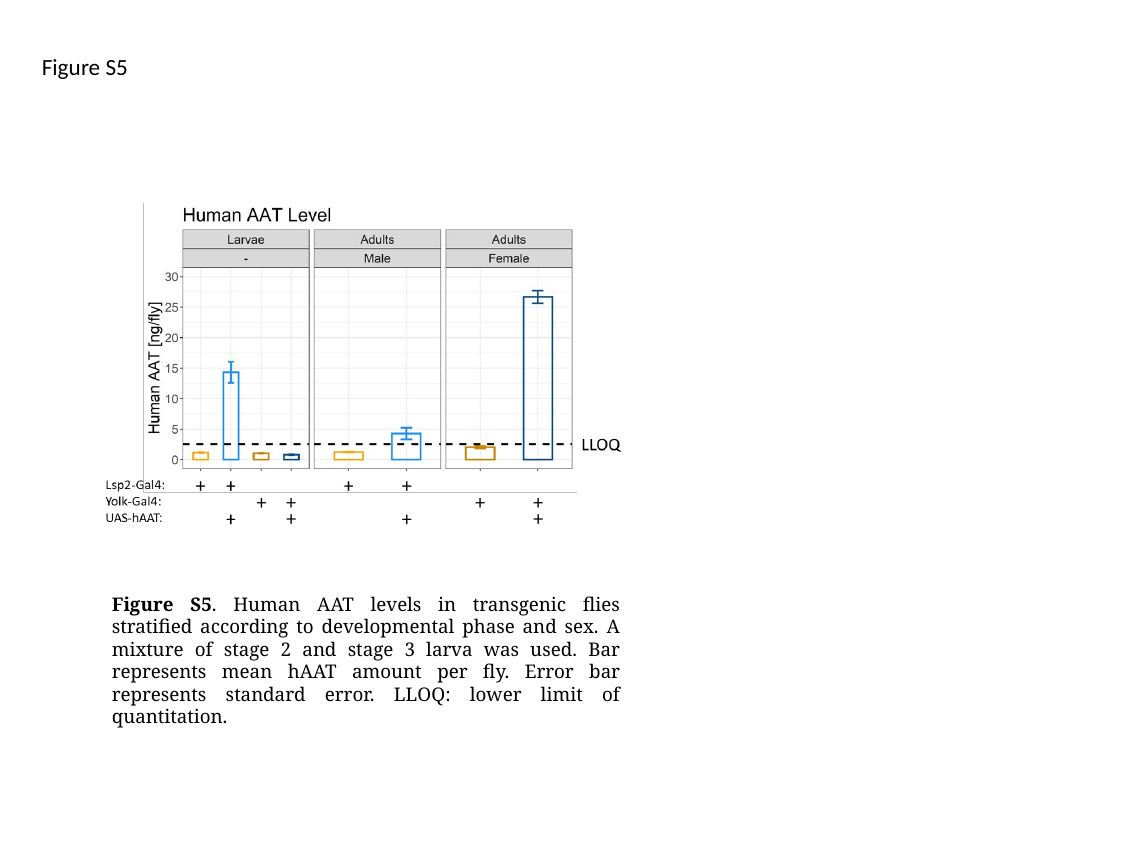

Figure S5
Figure S5. Human AAT levels in transgenic flies stratified according to developmental phase and sex. A mixture of stage 2 and stage 3 larva was used. Bar represents mean hAAT amount per fly. Error bar represents standard error. LLOQ: lower limit of quantitation.

## Slide 7
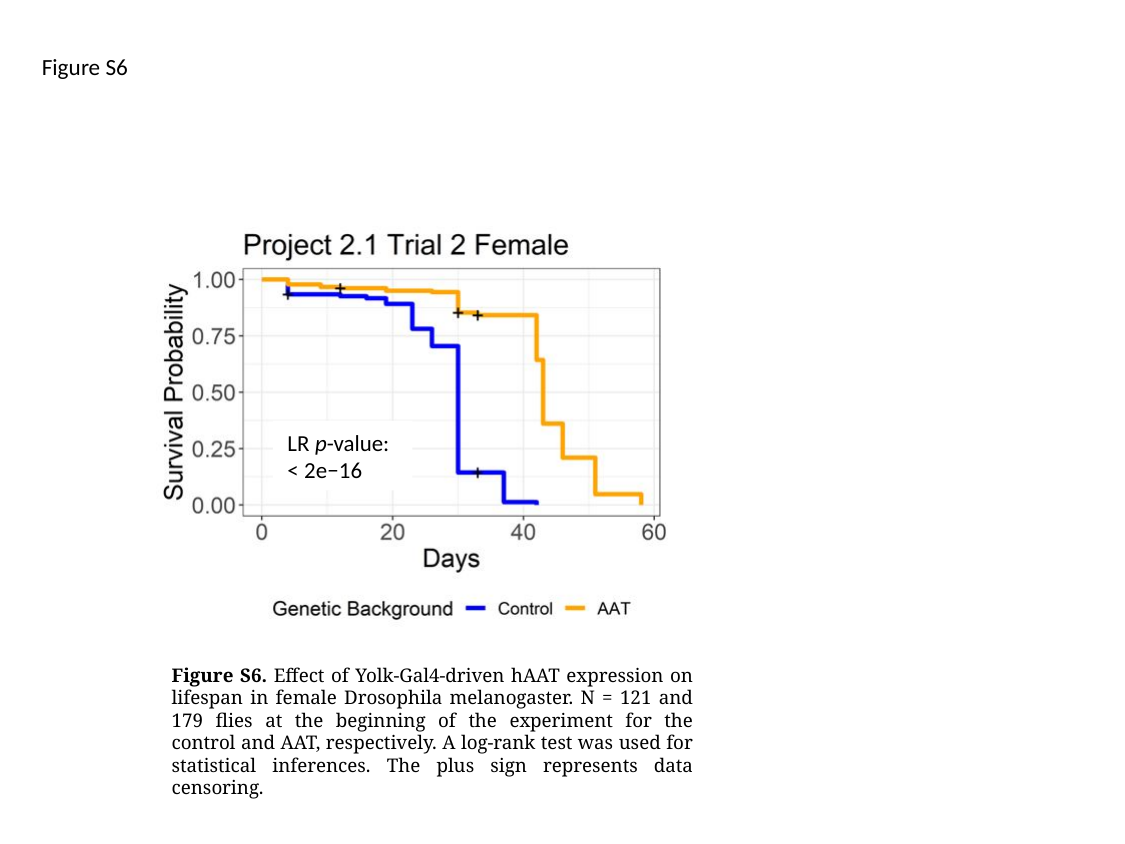

Figure S6
LR p-value:
< 2e−16
Figure S6. Effect of Yolk-Gal4-driven hAAT expression on lifespan in female Drosophila melanogaster. N = 121 and 179 flies at the beginning of the experiment for the control and AAT, respectively. A log-rank test was used for statistical inferences. The plus sign represents data censoring.
